# Orofacial myofunctional and anthropometric characteristics of children with and without microcephaly: a case-control study

**DOI:** 10.1590/1678-7757-2024-0473

**Published:** 2025-07-07

**Authors:** Andréa Monteiro Correia MEDEIROS, Gabriela Rodrigues Dourado NOBRE, Geyse do Espírito Santo REZENDE, Íkaro Daniel de Carvalho BARRETO, Jonan Emi Valencia CARDENAS, Sarah Catarina Santos NASCIMENTO, Anna Luiza dos Santos MATOS, Asenate Soares de Matos PEREIRA, Ricardo Queiroz GURGEL, Giédre BERRETIN-FELIX

**Affiliations:** 1 Universidade Federal de Sergipe Departamento de Fonoaudiologia São Cristóvão Sergipe Brasil Universidade Federal de Sergipe, Departamento de Fonoaudiologia, São Cristóvão, Sergipe, Brasil.; 2 Universidade Federal de Sergipe Programa de Pós-Graduação em Ciências da Saúde Departamento de Medicina Aracaju Sergipe Brasil Universidade Federal de Sergipe, Programa de Pós-Graduação em Ciências da Saúde, Departamento de Medicina, Aracaju, Sergipe, Brasil.; 3 Universidade Federal de Sergipe Programa de Pós-Graduação em Ciências Aplicadas à Saúde Lagarto Sergipe Brasil Universidade Federal de Sergipe, Programa de Pós-Graduação em Ciências Aplicadas à Saúde, Lagarto, Sergipe, Brasil.; 4 Universidade de São Paulo Faculdade de Odontologia de Bauru Departamento de Fonoaudiologia Bauru São Paulo Brasil Universidade de São Paulo, Faculdade de Odontologia de Bauru, Departamento de Fonoaudiologia, Bauru, São Paulo, Brasil.; 5 Fundação de Ensino e Pesquisa de Ciências da Saúde Escola Superior de Ciências da Saúde Brasília Distrito Federal Brasil Fundação de Ensino e Pesquisa de Ciências da Saúde, Escola Superior de Ciências da Saúde, Brasília, Distrito Federal, Brasil.; 6 Centro Brasileiro de Pesquisa em Avaliação e Seleção e de Promoção de Eventos Brasília Distrito Federal Brasil Centro Brasileiro de Pesquisa em Avaliação e Seleção e de Promoção de Eventos – CEBRASPE – Brasília, Distrito Federal, Brasil.

**Keywords:** Child, Preschool, Anthropometry, Microcephaly, Case-control studies

## Abstract

**Objective:**

This study aimed to describe and compare morphofunctional orofacial aspects between subjects with and without Zika virus-related microcephaly.

**Methodology:**

This was a descriptive, cross-sectional, case-control study with both qualitative and quantitative components. All subjects were born between 2015 and 2016, during the Zika virus outbreak in the Northeast region of Brazil. A total of 48 children were included: 24 with Zika-related microcephaly (MG) and 24 without the condition (CG). We performed the Preliminary Expanded Protocol of Orofacial Myofunctional Evaluation with Scores (OMES-E) for all subjects. Orofacial anthropometric measurements were obtained from 36 of the 48 participants, including 18 from the MG and 18 from the CG.

**Results:**

We found lower swallowing efficiency scores in children with microcephaly aged 13–18 months. Significant differences (p<.001) were found between the MG and CG for scores related to the face, cheeks, and total stomatognathic functions. When stratified by age group, differences (p<.001) were found in total scores between MG and CG subjects in the age groups up to 24 months. We found lower scores in the 13–18-month group with microcephaly for swallowing efficiency: 1.3 (SD: .8) versus 5.3 (SD: 1.2); and in the 19–24-month group; for bite: 1 (SD: 0) and 3.9 (SD: .3), and 1.9 (SD: 2.7) and 9.5 (SD: .9); in addition to facial changes: 9.8 (SD: 1.2) and 11.8 (SD: .6). Differences were found in anthropometric orofacial measurements for the upper third of the face (d=-1.215, p<.001) (MG<CG); proportion between the upper third/middle third (d=.463, p=.018) (MG<CG); and upper lip and philtrum (MG>CG) (d=-.679, p<.001).

**Conclusion:**

Subjects with microcephaly had altered orofacial myofunction, especially related to swallowing and chewing difficulties in early ages.

## Graphical abstract

**Figure:**
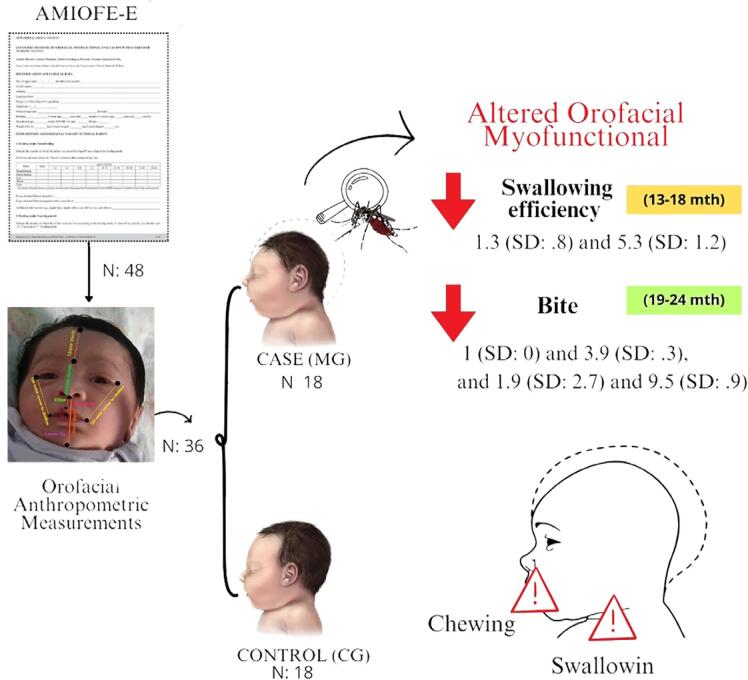

## Introduction

The emergence of Zika virus infection-related microcephaly cases since late 2015 was an alarming public health issue in Brazil and globally.^1-3^ During the 2015–2016 outbreak, the state of Sergipe reported 24.1 cases per 10,000 live births, with confirmed microcephaly cases in 26 of the 75 municipalities in the state.^4^

Congenital Zika Syndrome (CZS) is characterized by intracranial calcifications, ventriculomegaly, and reduced brain volume. Its occurrence requires transplacental transmission from a mother infected by the *Aedes* spp. mosquito or through sexual contact. There is a strong consensus that the Zika virus infection causes microcephaly and other neurological complications associated with CZS.^5^ In addition to microcephaly, affected individuals may present with delayed neuropsychomotor development, auditory and visual impairments, dyskinesia, hypertonia, hypotonia, hemiplegia, hemiparesis, spasticity and hyperreflexia,^6^ as well as craniofacial disproportions, spasticity, seizures, irritability and brainstem dysfunction, resulting in swallowing difficulties, limb contractures, and further auditory and ocular abnormalities.^7-8^ Common clinical signs in newborns include motor, cognitive, and perceptive impairments, such as progressively impaired sucking, swallowing, and breathing; inefficient sucking; uncoordinated tongue and jaw movements; downward weight-bearing curve; fatigue during feeding; and episodes of regurgitation or aspiration.^9-10^

A high prevalence of breastfeeding (BF) in the first hour of life has been observed in infants with Zika virus infection, ^11^ while the prevalence of exclusive BF up to sixth months of age remains low.^12^ The presence of multiple comorbidities directly interferes with sucking reflexes, leading to difficulties in sucking and swallowing during the sixth month of life.^11^ After three months, these difficulties tend to worsen, causing significant oral dysfunction, dystonic tongue movements, and pharyngeal hyposensitivity, increasing the risk of laryngotracheal aspiration, especially with liquids.^13^ Changes in orofacial motricity (OM) and in stomatognathic system (SS) functions have also been reported, such as poor lip sealing, predominant oronasal breathing, absence of chewing, and inefficient swallowing. Regarding infant dentition, studies indicate that microcephaly is a risk factor for changes in odontogenesis and enamel formation in the primary dentition.^15-17^

Aspects of OM during early childhood, the relationship between the craniofacial patterns and SS functions, and orofacial anthropometry^18^ should be investigated, as they complement clinical evaluation and are useful to objectively describe craniofacial variations. However, little is known about early- facial anthropometric measurements^19,20^in infants with microcephaly, and no previous studies have any orofacial myofunctional system-related anthropometric face measurements. At the time of the Zika virus outbreak, when many children were born with microcephaly, no standardized or validated instruments were available to assess their orofacial myofunctional condition, which was a challenge for clinicians and researchers.

Given the importance of investigating microcephaly-related aspects, this study aimed to describe and compare anthropometric orofacial characteristics and orofacial myofunctional patterns between patients with Zika-related microcephaly and those with no morbidities. We hypothesized that statistically significant differences would be found in most orofacial structures and functions between both groups.

## Methodology

### Study design and population

The study was conducted between May and December 2019 as part of a postdoctoral project in Sergipe, Brazil. Ethical approval was obtained from the Human Research Ethics Committee at CEP UFS (CAAE: 12529419.6.0000.5546), and the study was carried out according to the Declaration of Helsinki. Informed consent was obtained from all subjects’ parents/caregivers. This is a case-control, descriptive, cross-sectional study with a qualitative and quantitative approach. Participants were born in late 2015 and 2016, during the Zika virus outbreak in Northeastern Brazil.

The microcephaly group (MG) included children born and followed by the maternity clinic Nossa Senhora de Lourdes, in Sergipe, Brazil, with a Zika virus infection diagnosis and enrolled in the microcephaly follow-up protocol.^7^ The control group (CG) consisted of infants and preschoolers from a daycare center, paired with MG according to age group and gender. CG included children born under similar socio-demographic conditions as the MG, considered healthy following Zika-related microcephaly protocol, and with no signs of neuropathy that would warrant further investigation.

Exclusion criteria included functional/clinical instability, such as the need for respiratory support, which was considered incompatible with the data collected in this study or not authorized by the subject’s guardian. Additionally, infants with intense motor activity during the examination were excluded due to the risk of injury from the measuring instrument (caliper). The etiology of microcephaly in the study subjects was specifically associated with the Zika virus outbreak that caused microcephaly in Brazil in late 2015 and 2016, as shown by imaging tests (tomography, magnetic resonance, or ultrasonography) and serology (IgG +).

The Preliminary Expanded Protocol of Orofacial Myofunctional Evaluation with Scores (OMES-E) involved 48 subjects aged between seven and 32 months, of both genders, divided into groups with and without microcephaly, as shown in Table 1.

**Table 1:** Characterization of the studied population regarding Orofacial Motricity (Microcephaly Group and Control Group), according to age group and sex.

	Group	
	Microcephaly	Control
	(n=24)	(n=24)
**Age (Months), n (%)**		
07-12	1 (4.2)	1 (4.2)
13-18	8 (33.3)	8 (33.3)
19-24	13 (54.2)	13 (54.2)
25-32	2 (8.2)	2 (8.2)
**Sex, n (%)**		
Females	12 (50.0)	12 (50.0)
Males	12 (50.0)	12 (50.0)

Captions: n – absolute frequency. % – percent frequency.

The evaluation of anthropometric orofacial measurements included 36 subjects aged between 10 and 32 months, also of both genders, divided into groups with and without microcephaly, as shown in Table 2.

**Table 2:** Characterization of the studied population regarding the orofacial anthropometric measurements (Microcephaly Group and Control Group), according to age and gender.

	Group		
	Microcephaly	Control	p-value
	(n=18)	(n=18)	
**Age (Months), n (%)**			
07-12	1 (5.5)	1 (5.5)	0,980^CM^
13-18	2 (11.1)	2 (11.1)	
19-24	11 (61.1)	12 (66.7)	
25-32	4 (22.2)	3 (16.7)	
**Sex, n (%)**			
Females	8 (44.4)	8 (44.4)	1,000^F^
Males	10 (55.5)	10 (55.5)	

Captions: n – absolute frequency. % – percent frequency. CM Chi-Square Test with Monte-Carlo simulations. F Fisher Exact test.

### Data collection and recording procedures

The procedures applied to the MG have been previously described in the literature,^14^ and the information previously registered in the database was reorganized and comparatively analyzed against the CG. Some infants did not tolerate being evaluated alone in the assessment room due to discomfort with an unfamiliar individual or procedure. In such cases, the evaluation was conducted in the presence of a familiar adult—either the teacher (at the daycare center) or mother (at the outpatient clinic)—or with the subject sitting on the guardian’s lap. Regardless of the location or context of data collection during the orofacial myofunctional assessment, all assessments followed the same standardized protocol.

Preliminary Expanded Protocol of Orofacial Myofunctional Evaluation with Scores (OMES-E).There were no specific instruments validated in the OM field, despite the necessity to document the orofacial characteristics of infants with Zika-related microcephaly. The need to adapt the OMES-E Infants protocol became evident with the birth of individuals with microcephaly resulting from the Zika virus outbreak in Northeastern Brazil.

Individual clinical evaluation was performed using photographic records of orofacial structures and video footage of the feeding situation. Videos were recorded in MP4 format and photographs in JPEG. All records were subsequently reviewed to apply the protocol and perform the analysis for each subject. The same speech-language pathologist, who was properly trained and calibrated, analyzed the evaluation images and recorded the data in the Preliminary OMES-E Infants (age 6–24 months).^14^

The following aspects were analyzed: face (symmetry, proportion between facial thirds, and nasolabial fold); appearance of the cheeks (volume and tone); lips (function at rest, volume/configuration, labial commissures); mentalis muscle (contraction); tongue (position/appearance, volume); hard palate (width and height); breathing (mode); feeding (type of utensil used); deglutition: behavior of lips (sealing, contraction, and labial interposition), tongue (position within the oral cavity, interposition between teeth or along gingival margins); and other behaviors and signs of alteration (food escape, associated head and body movements, choking, repeated swallows for a single bite), efficiency (acceptance of different food consistencies—solid, pasty, and/or liquid); biting: mode of incision of the food, teeth used, absence of biting; chewing: type, whether bilateral (indicates the preferred side) or absence of food crushing.

The adapted version of the Preliminary OMES-E Infants^14^ initially had scores by functional blocks, with the maximum possible score ranging from 86 points for age groups up to 12 months, to 104 points for ages from 13 months onward. Higher scores correspond to a better orofacial myofunctional pattern, although this instrument does not establish a cut-off score to determine a normality pattern.

### Anthropometric orofacial measures evaluation

The orofacial anthropometric points were gently palpated on the subjects before measurements were taken and marked with a dermatographic pencil to guarantee location accuracy. The zeroing function of the digital caliper was verified, keeping the caliper fully closed until the displayed showed 0.00.

All measurements were obtained using the caliper rods for external measurement without pressing the tips (which were protected with micropore tape) against the skin surface. After each subject’s evaluation, the caliper rods were disinfected with hydrated ethyl alcohol for 30 seconds.

The following anthropometric measurements were taken as described in the literature:^20^ upper third of the face (tr-g); middle third of the face (g-sn); lower third of the face (sn-gn); distance between the outer corner of the eye and the right and left cheilion (ex-ch); philtrum height (sn-ls); upper lip height (sn-sto); and lower lip height (sto-gn).

Participants should remain still during measurements. Given their age group, agitation or sudden movements were a possible. Due to the behavioral characteristics of subjects with Zika-related microcephaly, there was a risk of irritability or sudden movements that could cause injuries from excessive manipulation during the measurement procedure. Therefore, all orofacial measurements were taken twice for each subject by the same evaluator in immediate succession. Since there are no established thresholds in the literature for technical measurement error in orofacial anthropometry, the Bland–Altman plot was used to assess possible discrepancies. It was observed, that for the variables analyzed, discards were justified on one or two occasions per variable, resulting in at least 95.6% of the observations demonstrating reliability.^20^

Anthropometric orofacial measurements were collected from a frontal view and recorded in millimeters during the examination according to the data collection protocol.^20^ Subsequently, the arithmetic mean of the two measurements (I and II) for each structure was calculated.

### Instruments/materials used

Protocols: Preliminary OMES-E Infants (6–24 Months), adapted for infants,^14^ and the protocol for recording anthropometric orofacial measurements.^20^

Materials for general physical exams: Tongue depressor, procedure gloves, cotton, 70% ethyl alcohol, and dermatographic pencil (black Make B Eye Pencil).

Digital caliper: A TTC Stainless Hardened stainless steel digital caliper with a liquid crystal display, millimeter unit system, 0.01 mm resolution, and ± 0.03 mm/0.001 mm accuracy was used. The caliper tips were coated with adhesive tape as a safety procedure to avoid injuring the infants.^20^

Food/utensils: Liquids (water or milk); pasty/smashed foods (puree and banana); and solids (cream cracker, rice, beans, and meat). Utensils included baby bottle, cup, plate, and spoon/fork.

Feeding methods: Infants in the MG and younger participants in the CG were fed by their guardians, as they were unable to feed themselves. Older CG participants fed themselves. We offered the amount of food the infant usually accepted, as informed by the guardian and/or teacher. All infants were fed orally, and none used an orogastric tube. Liquid and pasty food swallowing evaluation was performed in infants from six months of age. Solid food swallowing and chewing evaluations were performed from 12 months of age. However, some infants were not offered certain textures if their guardians or teachers reported they would refuse them.

Without specific material, the International Dysphagia Diet Standardisation Initiative (IDDSI)^21^ framework scale was used to classify the diet textures addressed in the Preliminary OMES-E Infants (6–24 Months) and to standardize the results.

In general, we evaluated liquid feeding by offering 50 to 100 ml, depending on the child’s ability to coordinate suction, swallowing, and breathing, as determined by the guardian or teacher’s report, using either a bottle or cup. We evaluated the pasty/smashed food feeding using a small portion equivalent to a mashed banana, with an average offering of two to five spoonfuls. We preferably evaluated solid food feeding using one cream cracker biscuit or a small shallow dish containing typical solid foods, corresponding to approximately 100 to 150 g, which corresponds to one to three bites. We offered food consistencies respecting the child’s ability and coordination (swallowing/chewing). We interrupted food supply whenever significant swallowing incoordination was observed, such as escape of more than half of the offered food, choking, or coughing. If the infant did not accept a specific food texture, they received the lowest possible score.

### Data analysis methodology

Data were described using simple and percentage frequencies for categorical variables (OMES-E adapted for infants) and mean and standard deviation for continuous variables (anthropometric orofacial measurements).

Associations were tested using Fisher’s exact test, Pearson’s chi-square test, and Pearson’s chi-square with Monte Carlo experiments.^22^ Mean differences between independent groups were tested using the Mann–Whitney test (not parametric) or the independent t-test (parametric),^23^ depending on the verification of the normality assumption by means of the Shapiro–Wilk test.^24^

Correlations between anthropometric measurements were assessed using Pearson’s (when normal) or Spearman’s (when not normal) correlations.^25^ Differences in anthropometric measurements were quantified using Cohen’s d effect sizes (when normal) or rank-biserial correlation (when not normal).^26^ The significance level was set at 5%, and the software used was R Core Team 2019.

## Results

### Orofacial myofunctional evaluation: Preliminary OMES-E infants (6–24 Months)

Forty-eight subjects participated, divided into groups with and without morbidity (microcephaly). MG: 24 subjects with microcephaly, including 12 females (50%) and 12 males (50%), with a minimum age of seven months and a maximum age of 32 months (median of 19 months). The MG was composed after active search, and the number of participants was characterized by a convenience sample. Among subjects with microcephaly (N=24), 70% (N=17) had imaging findings such as calcifications, lissencephaly, and ventriculomegaly; 4.2% (N=1) were diagnosed by IgG + serology; 12.5% (N=3) had both imaging and serology alterations; and 12.5% (N=3) were considered undefined (cases under investigation). CG: 24 subjects without microcephaly, matched for age and gender with the MG in a 1:1 ratio, appropriate for the statistical analyses performed.

When all cases were analyzed together, significant differences (p<0.001) were found between groups in the scores for face, cheeks, and total stomatognathic functions. Table 3 shows the results, providing a comprehensive overview.

**Table 3:** Distribution of study participants by group (with and without microcephaly), according to age group, sex, and scores obtained with Preliminary OMES-E Infants.

	Group		
	Microcephaly	Control	p-value
	(n=24)	(n=24)	
**Age (Month)**, n (%)			
07-12	1 (4.2)	1 (4.2)	1,000^CM^
13-18	8 (33.3)	8 (33.3)	
19-24	13 (54.2)	13 (54.2)	
**25-32**	2 (8.2)	2 (8.2)	
**Sex**, n (%)			
Females	12 (50.0)	12 (50.0)	1,000^F^
Males	12 (50.0)	12 (50.0)	
**OMES-E**, Mean (SD)			
Face	9.6 (1.5)	11.8 (0.5)	<0,001^W^
Cheeks Appearance	6.9 (0.9)	7.9 (0.4)	<0,001^W^
Lips	10.3 (1)	11.1 (1.3)	0,003^W^
Mental Muscle	3.6 (0.6)	3.6 (0.6)	0,645^W^
Tongue	7.5 (1.2)	7.9 (0.6)	0,173^W^
Hard Palate	7.2 (1.3)	7.6 (1)	0,173^W^
Breathing	2.8 (0.7)	3.8 (0.5)	<0,001^W^
Deglutition: Lips behaviour	1.6 (1.6)	5 (1.4)	<0,001^W^
Deglutition: Tongue behavior	2.7 (1.2)	4 (0.2)	<0,001^W^
Deglutition: Other behaviour and alteration signs	10.5 (1.2)	11.9 (0.4)	<0,001^W^
Deglutition: Efficiency	1.8 (1)	5.1 (1.3)	<0,001^W^
Bite	1 (0)	3.8 (0.6)	<0,001^W^
Chewing	2.3 (3.1)	7.8 (3.3)	<0,001^W^
Chewing: Other behaviours and alteration signs	3.7 (0.6)	5.5 (1.7)	0,001^W^
Total Score	62.6 (12.7)	96.8 (6.1)	<0,001^W^

Captions: n – absolute frequency. % – percent frequency. SD – Standard Deviation. CM Chi-Square Test with Monte-Carlo simulation. F – Fisher Exact Test. W – Mann-Whitney Test.

When analyzing the subjects by age group, differences (p<0.001) were found between infants in the MG and CG for the total score, as shown in Table 4, as well as in specific aspects, which are detailed throughout the text.

**Table 4:** Distribution of Infants (with and without microcephaly), according to total scores obtained with Preliminary OMES-E Infants.

Total Score	Group				
	Microcephaly		Control		p-value
OMES-E	Mean (SD)	n	Mean (SD)	n	
13-18 months	63.6 (9.7)	8	94.5 (6.9)	8	<0,001
19-24 months	61.6 (14.3)	13	97.8 (5)	13	<0,001
25-32 months	60 (21.2)	2	104 (0)	2	0,333

Captions: SD – Standard Deviation. Mann-Whitney Test. n – absolute frequency.

Differences (p<0.001) were found between infants in the MG and CG, with lower scores observed in the former: 1.3 (SD: 0.8) in the MG and 5.3 (SD: 1.2) in the CG for swallowing efficiency at earlier ages (13–18 months). This difference was not observed in the older infant group (19–24 months). Among infants aged 19 to 24 months, lower scores were again observed in the MG in aspects related to biting: 1 (SD: 0) versus 3.9 (SD: 0.3) in the CG; and chewing: 1.9 (SD: 2.7) in the MG versus 9.5 (SD: 0.9) in the CG. Additionally, differences were also noted in facial aspects, with scores of 9.8 (1.2) in the CG and 11.8 (0.6) in the MG.

Among preschoolers (25–32 months), no significant differences were found between MG and CG. However, lower scores were noted in the MG for swallowing-related aspects (lip and tongue behavior; efficiency) and biting.

### Anthropometric orofacial measurements

Thirty-six subjects were divided into two groups with a minimum age of 10 months, a maximum age of 32 months, and a median age of 21.5 (SD: 4.6). The MG consisted of 18 subjects with microcephaly who also underwent orofacial myofunctional assessment. The CG included 18 subjects without microcephaly, matched 1:1 with the MG based on age, gender, and birth region, in accordance with the requirements for statistical analysis.

Anthropometric measurements are shown in Table 5. Differences were found between the MG CG for the Upper Third of the Face (MG<CG); Proportion between the Upper Third/Middle Third (MG<CG); and Upper Lip and Philtrum (MG>CG).

**Table 5:** Distribution of study participants by group (with and without microcephaly), according to the anthropometric orofacial measurements obtained.

	Microcephaly	Control	p-value	E	R
	(n=18)	(n=18)			
	Mean (SD)	Mean (SD)			
Upper Third	39.69 (5.36)	46.61 (6.02)	**<0,001**^**T**^	-1,215^D^	0,950^P^
Medium Third	39.84 (6.31)	39.51 (2.55)	0,840^T^	0,068^D^	0,917^P^
Lower Third	49.63 (5.56)	49.10 (4.09)	0,746^T^	0,109^D^	0,956^P^
Outer corner of right eye	52.22 (5.52)	52.39 (3.57)	0,914^T^	-0,036^D^	0,934^P^
Outer corner of left eye	52.03 (4.60)	51.76 (3.79)	0,846^T^	0,065^D^	0,962^P^
Upper Lip	17.41 (3.12)	15.34 (1.97)	**0,018**^**W**^	0,463^B^	0,754^S^
Lower Lip	33.01 (4.92)	31.05 (4.13)	0,203^T^	0,302^D^	0,956^P^
Filter	11.57 (2.21)	9.39 (2.60)	**0,011**^**T**^	0,500^D^	0,977^P^
Upper Lip/Lower Lip	0.54 (0.14)	0.50 (0.08)	0,111^W^	0,315^B^	
Upper Third/Medium Third	1.01 (0.13)	1.18 (0.14)	**<0,001**^**W**^	-0,679^B^	
Medium Third/Lower Third	0.81 (0.10)	0.81 (0.07)	0,892^T^	0,025^D^	

Captions: SD – Standard Deviation. T Unpaired T-Test. W Mann-Whitney Test. E – Size Effect. D Cohen’s D. B Rank Biserial Correlation. R – Correlation between measures. P Pearson Correlation. S Spearman Correlation. Significant results (p<0,05) in bold.

## Discussion

This study design enabled an equal distribution of participants with and without microcephaly regarding age and gender. Participants ranged from seven to 32 months of age, with an average of 19.5 months (SD: 4.8), making it possible to assess OM aspects during the first two years of life.

This study used the preliminary version of the OMES-E Infants protocol,^14^ which was later validated and published. One difference between the preliminary protocol used in this study and the validated protocol^14^ lies in the adaptation of certain terminology and structural adjustments. In the table related to facial assessment, specifically the appearance of cheeks and lips, terminological modifications were made for greater clarity and standardization. Additionally, the evaluation table for the mentalis muscle was excluded, while items assessing the frenulum and its characteristics were added to the tongue assessment table. Another modification was the inclusion of a specific table to assess the soft palate and uvula, expanding the scope of the orofacial myofunctional analysis. Finally, adjustments were made to the scoring criteria in the functional blocks table to refine the calculation of the total score, enhancing precision in the result interpretation. These differences reflect the advances made during the protocol validation process, aiming to improve its applicability and methodological robustness, while also contributing to greater reliability in future analyses.

### Orofacial myofunctional assessment

The results of the orofacial myofunctional evaluation of the 48 study participants show important differences between groups. Consistently lower values were observed in the MG for both orofacial myofunctional structures (face and cheeks) and all SS functions, consistent with previous studies about the characterization and development of individuals with CZS.^5,7^

Joint analyses of cases showed statistical differences in the MG when compared to the CG, with notably poorer scores in facial morphology, cheek appearance, breathing, swallowing (lip behavior, tongue, and signs of dysfunction), swallowing efficiency, biting and chewing, and total function scores. These aspects could be considered potential risk factors for compromised feeding performance,^11,27^ delayed orofacial myofunctional system development,^18^ and dysphagia.^13^

The significant differences for face and cheek aspects in this study, with lower scores in the MG, are consistent with a previous study^14^ that reported altered facial and cheek appearance in infants with microcephaly. In that study, the lower third of the face increased slightly compared to other facial thirds in 87.5% of cases, while cheek tension and configuration were normal in only 41.7% of cases.^14^ It is believed that reduced cheek tension is linked to a lower prevalence of breastfeeding or the presence of sucking difficulties.^11-12^ The increased proportion of the lower third of the face may be related to oral breathing patterns.^28^

Among preschoolers (25–32 months), no significant differences were found between the MG and CG, despite lower scores related to biting swallowing in the former. The absence of significantly different values may be attributed to the small number of preschoolers (N=4) analyzed within this age range.

This study found that breathing-related scores also had significant differences between groups and was lower in the MG. Inefficient nasal breathing warrants special attention, since breathing with closed lips is fundamental for adequate bucco-facial and neuromuscular development.^28^ Oral breathing is a risk factor for proper orofacial myofunctional system development, especially in the population with Zika-related microcephaly, who frequently present with delayed neuropsychomotor development and craniofacial disproportion.^5^

A pattern of lip and tongue behavior during swallowing was observed with significantly lower values in the MG, as found in a previous study^28^ involving mouth-breathing individuals without neurological conditions. Conversely, other authors^29^ have described poor oral motor control, including frequently parted lips and tongue protrusion, as aspects inherent to neurological issues in children and adolescents.

It should be noted that the evaluation of chewing and swallowing employed a variety of utensils and food consistencies, with standardization adapted to the participant’s age group and respecting each child’s individual acceptance, considering the particularities of the population affected by the Zika virus. The procedure followed the recommendations of the protocol, which emphasizes the importance of considering the child’s acceptance and aligning the evaluation with both age-appropriate expectations and the infant’s habitual eating patterns. Specifically regarding chewing, the protocol establishes that assessment should be based on the infant’s chronological age and developmental stage. Thus, the observed heterogeneity is not considered to have compromised methodological consistency, since the protocol guidelines were strictly followed.

In the analysis by age group, the MG presented poorer scores in swallowing efficiency during earlier ages (13–18 months), a difference not found in older infants (19–24 months). There were differences in the scores among infants between 19 and 24 months (lower in the population with microcephaly) between groups for aspects related to biting, chewing, and facial changes—findings consistent with previously discussed difficulties in orofacial structures. These results suggest that, in the population with microcephaly, early difficulties are more related to swallowing efficiency, while changes in biting and chewing become more evident at older ages, reflecting the child’s development. This result is consistent with a study^30^ that also reported swallowing difficulties in 78% of children with microcephaly under 24 months of age, whose main feeding difficulty was aspiration.

It is important to emphasize that teeth eruption facilitates the development of an efficient chewing pattern, which positively influences dental development. However, lower scores in the MG reveal an inadequate pattern for the development of infant feeding skills.^31^ Differences were found not only in oral structures—such as reduced number of teeth—but also in functional aspects, including swallowing and chewing patterns, with poorer scores in the MG. These findings underscore the impact of the form-function interrelationship and highlight the influence of microcephaly on dental development.^15 -17^

Neurological issues could also influence the orofacial myofunctional pattern in this population. Clinically, although the presence of orofacial structures influences orofacial function skills, we consider that such presence does not guarantee proper function, because they also depend on appropriate neuropsychomotor development.^32^ The lower scores obtained for swallowing and chewing in the MG corroborate previous findings,^14^ in which 100% of infants with microcephaly, regardless of age, did not exhibit biting behaviors. Furthermore, 83.3% did not perform grinding movements during chewing. Of those who accepted solid foods, 16.7% performed simultaneous bilateral chewing with a kneading pattern.^14^

Chewing absence in both groups after 12 months of age is concerning, as chewing is considered one of the most important SS functions. Grinding and correctly preparing the bolus during the oral phase favors swallowing and provides better food digestion and, consequently, improved nutritional outcomes.^33^ This study found differences across all age groups, with lower total scores in the MG, indicating a more compromised orofacial myofunctional pattern. These findings are in line with previous studies, ^14,29,34^which state that neurological limitations can cause several food-related challenges. Although the methodology adopted standardization to give the lowest possible score in cases of refusal of specific food textures, this does not necessarily confirm the presence of OM changes. Instead, it may reflect a greater risk for orofacial myofunctional disorder.

Food refusal is an eating disorder that can emerge during the infant feeding process and is frequently observed in pediatric populations. Broadly, feeding disorders refer to difficulties in developing the skills necessary for safe and effective eating and drinking. Considering that orofacial motricity encompasses fundamental skills for adequate feeding performance, the presence of eating difficulties, especially food refusal, should be interpreted as a warning sign for a possible orofacial myofunctional disorder.^35^

The significant structural (face, cheeks) and functional (swallowing, 13–18 months; and chewing, 19–24 months) differences observed between groups support findings reported by the Brazilian Ministry of Health.^9^ These data highlight that CZS can cause feeding difficulties, including oral motor incoordination during swallowing and sucking, difficulty coordinating these functions with breathing, and episodes of gastroesophageal reflux. As a result, affected children often exhibit food refusal and are at heightened risk of developing malnutrition.^9,30^

Feeding difficulties commonly observed in this population are aggravated by brain malformations and injuries that affect the central control of swallowing, chewing, and sucking. This increases the risk of structural alterations, leading to orofacial inadequacies that compromise swallowing in early infancy and impact chewing throughout development.

Therefore, our findings reveal important orofacial myofunctional challenges with clinical implications across the SS throughout early development, indicating the need to establish a continuous therapeutic proposal and follow-up of Zika virus-related microcephaly effects on patients and their families.

### Anthropometric orofacial measurements

Although this study is unprecedented, as no previous studies on anthropometric measurements in infants and preschoolers with CZS were found, it refines the theoretical framework adapted by Medeiros, et al.^20^ (2019), which outlined anthropometric points and lines to be trained in neonates.

The statistical differences found between groups revealed smaller measurements in the MG for the upper third of the face (MG<GC) and in the proportion between upper third/middle third (MG<GC). This corroborates a previous study,^14^ in which the proportion between the thirds of the face was altered in most cases with microcephaly (87.5%), with the lower third slightly enlarged in relation to the other thirds.^14^ These findings are consistent with the known craniofacial alterations associated with microcephaly, a congenital malformation marked by disproportionate and inadequate brain development.^36^

In this sense, statistical analyses showed that microcephaly preserves facial bone structures regarding the distances between the outer corners of the eyes and the right and left lip commissures, maintaining facial symmetry. Even the measurements that showed differences in the upper lip and philtrum (MG>CG) can be considered minimal (approximately with an average of 2 mm), and therefore not clinically significant.

The results did not confirm the initial hypothesis of this study, which predicted significant statistical differences in all or most orofacial structures of infants with and without microcephaly. The absence of these differences may be explained by the fact that microcephaly is a malformation that mainly affects the upper region of the face and skull. Oral malformations are not usually present at birth or in earlier ages, as changes in these structures tend to be related to SS function disorders that occur later during development. It is worth noting that anthropometric orofacial measurements were taken at an early age. There is a recognized risk that changes in orofacial structures may emerge over time, since the swallowing and chewing difficulties evidenced in the case-control study presented in this research could, in the medium and long term, unfavorably impact the harmonic growth of phonoarticulatory organs.

Although no significant structural changes were found in oral measurements, longitudinal studies are needed to monitor the craniofacial development of this population, especially considering changes in stomatognathic structures and functions identified during the speech-language assessment. The orofacial and anthropometric myofunctional characterization of infants and preschoolers with CZS highlights the importance of continuous therapeutic proposals and monitoring the impact of microcephaly caused by the Zika virus. Further research using anthropometric measurements is essential to develop evaluation protocols for this age group.

### Limitations

We intended to collect a greater amount of orofacial anthropometric data from individuals with Zika-related microcephaly; however, the number of participants was limited due to the reduction in births with this malformation and the high motor activity of some infants, which prevented the inclusion of their measurements. Despite the challenge of measuring participants, especially those with microcephaly, a strategy of repeated anthropometric measurement was used to avoid discrepancy or bias in data collection. This approach, which involved measuring the same region twice, was followed in accordance with the recommended procedure.^20^

The scarcity of studies and protocols for infants and preschoolers with CZS made comparisons difficult. To address this gap, this study proposed an adapted preliminary instrument, resulting in the content and appearance validation of the OMES-E Infants.^14^

The lack of specific instruments for this developmental age group was a relevant limitation; however, it met the urgent need to investigate a population affected by the Zika virus outbreak. For this reason, a preliminary version of the OMES-E Infants^14^ protocol was used, whose items closely resembled those in the later fully validated version, which achieved a high content validity index. This aspect partially mitigates the limitation, contributing to future research. The scarcity of validated instruments for the orofacial myofunctional evaluation of infants with microcephaly at the time motivated adaptations, such as those performed in the MMBGR protocol for infants and preschoolers,^37^ followed by rigorous validation procedures. It is noteworthy that the field has since advanced scientifically, and the selection of well-recognized instruments in Speech-Language Pathology—appropriately adapted and validated—offers robust methodological support, favoring comparisons between different populations in subsequent investigations.

Despite some methodological difficulties, careful attention was given to data collection and standardized application of the instrument in both groups.

## Conclusions

This study found significant differences in the orofacial myofunctional patterns and anthropometric orofacial measurements between subjects with Zika-related microcephaly and those without the impairment. Specifically, individuals with microcephaly showed worse orofacial myofunctional outcomes in the face, cheeks, total stomatognathic functions, and total scores for age groups up to 24 months. Significant impairments were noted in swallowing efficiency for the 13–18 and 19–24 months age groups, as well as in biting and facial changes. The MG exhibited smaller measurements in the upper third of the face and the proportion between the upper third/middle third, alongside greater measurements of the upper lip and philtrum.
